# The Impact of Fracture Liaison Services on Outcomes in Older Patients: A Systematic Review

**DOI:** 10.7759/cureus.95685

**Published:** 2025-10-29

**Authors:** Raghavee Neupane, Brogan Ness, Brahadesh Sivakumar, Stephanie Nagy, Marc M Kesselman

**Affiliations:** 1 Medicine, Nova Southeastern University Dr. Kiran C. Patel College of Osteopathic Medicine, Davie, USA; 2 Rheumatology, Nova Southeastern University Dr. Kiran C. Patel College of Osteopathic Medicine, Davie, USA

**Keywords:** fls, fracture liaison services, healthcare outcomes, multidisciplinary programs, older adults, osteoporosis, osteoporotic fractures, osteoporotic medication initiation, post-fracture care, refracture risk

## Abstract

Osteoporotic fractures are fractures caused by underlying osteoporosis that occur after minimal trauma. They represent a prevalent public health problem worldwide with significant economic burden and negative effects on quality of life. Fracture liaison services (FLS) are multidisciplinary programs aimed to decrease secondary fractures in high-risk patients by improving the post-fracture care gap. This review analyzes the impact of FLS on patient outcomes, especially refracture risk and osteoporosis medication initiation. This review was conducted using Ovid (MEDLINE), CINAHL, and Web of Science. Randomized controlled trials (RCTs), cross-sectional studies, observational studies, longitudinal studies, and cohort prospective/retrospective studies published between 2010 and 2024 were included. Selected articles also had a focus on refracture risk and/or osteoporotic medication initiation, a patient cohort mean age greater than 65 years old, and patients presenting with an initial osteoporotic fracture (as opposed to a history of fractures). Out of 727 total articles screened, 49 were included. The most prescribed medications were zoledronic acid, denosumab, and alendronate. FLS implementation was generally associated with a decrease in refracture rate, but only 11 out of 20 studies that assessed this found a statistically significant difference. Variability in refracture rates among studies may be due to differences in program intensity and patient demographics (namely, age and level of function). FLS can be an effective model to target the post-fracture care gap in older osteoporosis patients and improve outcomes, particularly medication initiation. Future studies should evaluate best practices for FLS programs.

## Introduction and background

Osteoporosis is a metabolic bone disorder characterized by low bone mineral density (BMD) and altered microarchitecture, leading to increased risk of fragility fractures [1]. Hip fractures, in particular, have been shown to be associated with high rates of morbidity and mortality [1]. Despite this, many individuals with osteoporosis remain undiagnosed and undertreated. In the United States, the age-adjusted prevalence of osteoporosis among adults over 50 years was 12.6% between 2017 and 2018 [2]. Postmenopausal women are at a disproportionately higher risk of osteoporosis due to estrogen deficiency, which accelerates bone loss [3]. Approximately 50% of women and 20% of men will experience an osteoporosis-related fracture within their lifetime. Gender differences in the risk of osteoporotic fractures have been linked to several contributing factors. Specifically, women have a younger onset of bone loss and lose bone at a faster rate as compared to their male counterparts [4]. In addition, women experience a more significant decline in estrogen levels at a younger age due to menopause as compared to men, despite men also experiencing estrogen loss [4]. Furthermore, women have smaller bone size and a higher fall risk, predisposing them to fractures [4]. A prior low-trauma fracture roughly doubles the risk of future fractures in both sexes [5].

Fragility fractures are largely preventable with adequate screening, timely treatment, and lifestyle interventions. However, persistent care gaps leave many high-risk patients without appropriate therapy. To address this, fracture liaison services (FLS) have emerged as coordinated, multidisciplinary programs that identify patients with fractures, assess bone health, initiate secondary prevention (e.g., pharmacotherapy), and provide follow-up care and education [5]. Studies have shown that osteoporosis and the associated risk of fractures can be prevented with increased screening and preventative lifestyle strategies. This review aims to understand whether the implementation of specialized programs to reduce fractures can reduce morbidity and mortality tied to fragility fractures and improve patient outcomes.

Osteoporosis develops gradually as the equilibrium between bone resorption and formation in bone remodeling becomes disrupted [6]. Bone remodeling occurs when bone cells called osteoclasts are activated and secrete acid at the bone surface to dissolve mineralized bone. The osteoclasts then undergo apoptosis and osteoblasts, bone-forming cells, and secrete bone matrix, which will later mineralize [7,8]. Bone remodeling is a continuous process, but in osteoporosis, bone resorption exceeds bone formation, resulting in progressive bone loss and a weakened skeletal structure [6]. Risk factors include age, gender, family history, hormonal changes, nutritional deficiencies (specifically calcium and vitamin D), genetic predisposition, smoking, excessive alcohol consumption, and specific medications [6,9]. Newer research has demonstrated that various chemokines, cytokines, and other immune system components play a role in regulating bone remodeling [8]. Estrogen deficiency in menopause promotes the secretion of and responsiveness to immune signaling molecules, such as tumor necrosis factor alpha (TNF-alpha) and interleukin-1 (IL-1), which stimulate osteoclastogenesis [7]. A lack of estrogen also enhances the effect of receptor activator of NF-κB ligand (RANKL), a molecule secreted by osteoblasts that promotes osteoclast survival, activation, and differentiation [7]. Early osteoporosis is often asymptomatic, but as the condition progresses, individuals may experience bone pain, height loss, and a stooped posture [6]. The hallmark of osteoporosis is fragility fractures, which occur with minimal trauma or falls [6].

The current standard for treating osteoporosis involves bisphosphonates, which induce osteoclast apoptosis and reduce bone resorption [10]. A 2023 meta-analysis showed a 32% reduction in clinical fractures with one to three years of bisphosphonate use and a 21% reduction with three to five years of bisphosphonate use as compared with placebo [11]. Meanwhile, the efficacy of bisphosphonates declines after five years of oral treatment or three years of intravenous therapy. This has led to recommendations for a "drug holiday" to lower the risks of atypical femur fractures [12].

Other treatment options include selective estrogen receptor modulators (SERMs), such as tamoxifen, and hormone replacement therapy (HRT), both of which may be associated with significant adverse effects, including an increased risk of venous thromboembolism. Newer agents, such as denosumab, can increase the risk of hypocalcemia and, in rare cases, osteonecrosis of the jaw. Teriparatide carries a potential risk of hypercalcemia and a theoretical risk of osteosarcoma with long-term use [6]. SERMs act as estrogen receptor agonists in bone, improving BMD and decreasing bone loss [7,13]. Denosumab is a monoclonal antibody (mAb) against RANKL that downregulates osteoclastogenesis [6]. Teriparatide, a synthetic analogue of parathyroid hormone (PTH), works as an osteoanabolic drug that stimulates osteoblast activity to promote bone formation [6]. While continuous PTH is classically associated with bone catabolism, intermittent dosing enhances bone anabolism [13,14].

Aside from medication, preventive measures, such as a diet rich in calcium and vitamin D, regular weight-bearing exercise, smoking cessation, and alcohol moderation, are key. Modifying the environment, including home safety adjustments and balance training, further reduces fall risks.

Osteoporosis-related fractures have been shown to be associated with significant physical, functional, and psychological consequences [15]. Globally, one in three women and one in five men over the age of 50 years will experience an osteoporotic fracture, with hip fractures being particularly debilitating [13]. Among patients with hip fractures, approximately 22% will die within one year due to complications [16], and only 33% of senior women regain their independence for basic daily activities [17]. The rate of refracture is highest within the first year and remains elevated for up to 25 years, especially for hip and vertebral fractures [18]. In this manuscript, refracture and secondary fracture are used interchangeably to describe any new fracture occurring at a previously affected site after initial healing.

Studies show that only 30% of patients who suffer an initial fragility fracture receive osteoporosis evaluation or treatment, making them three to four times more likely to suffer a subsequent fracture [19,20]. Fewer than one-third of patients with fractures adhere to prescribed osteoporosis medications long term [21], and one-fourth of the patients with fragility fractures received a DXA scan in the last two years before their fracture [22]. These gaps highlight systemic failures in continuity of care. The economic burden of osteoporosis is also substantial. The annual cost to the US healthcare system due to osteoporosis and related fractures exceeds $25 billion and is projected to rise further [23].

By standardizing post-fracture management, FLS aims to reduce secondary fracture risk, improve patient outcomes, and lower healthcare costs [5]. Typical FLS teams often include physicians, nurses, physiotherapists, dietitians, and administrative coordinators who work together to optimize post-fracture management [24]. Although the number of FLS programs in the United States is not consistently tracked, the American Orthopedic Association identified 240 established FLS programs in 2018. This review analyzes the impact of the FLS model on patient outcomes, focusing on morbidity rates, secondary fractures, hospital stays, initiation of anti-fracture medications, and quality of life.

## Review

Methods

A systematic literature review was performed using Ovid (MEDLINE), CINAHL, and Web of Science using the search terms ("Fracture Liaison Services") AND (“osteoporotic fractures” OR “osteoporosis”). To ensure the recency of the articles, only articles published between 2010 and 2024 were assessed. The articles were analyzed in a step-wise process by evaluating the title and abstract for relevance and then assessing the full-text manuscript. The Nova Southeastern University (NSU) library database was utilized to access databases and full-text articles.

**Figure 1:**
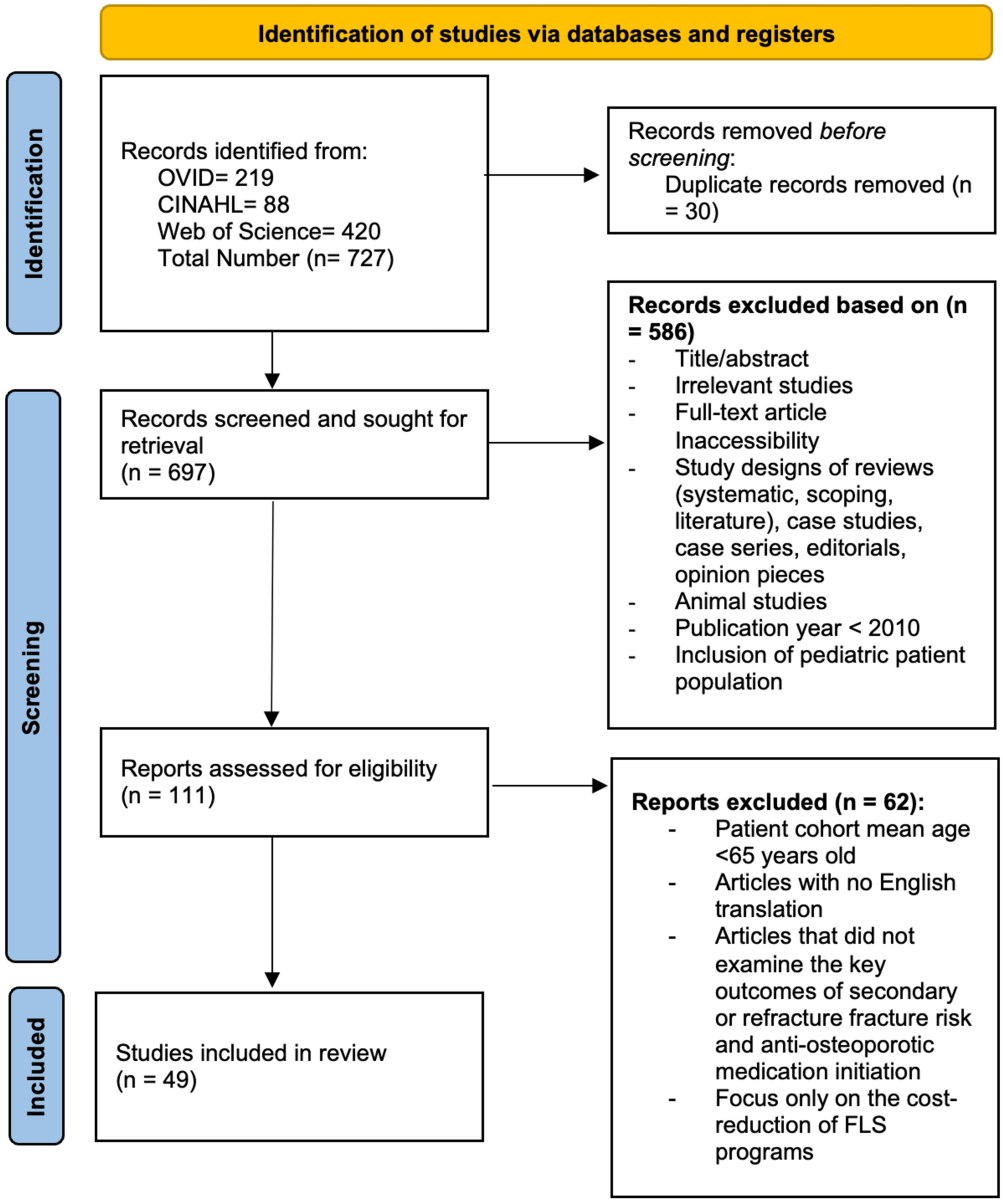
Preferred Reporting Items for Systematic Reviews and Meta-Analyses (PRISMA) Indicating Data Selection

For this review, randomized controlled trials (RCTs), cross-sectional studies, observational studies, longitudinal studies, and cohort prospective/retrospective studies were included. The population included patients with an average age above 65 years of age with an initial osteoporotic fracture. The outcome being observed is whether the initiation of FLS improved refracture or secondary fracture risk and osteoporotic medication initiation. Studies excluded from this review were literature, systematic or scoping reviews, animal studies, case studies and series, and editorials. Articles were excluded if the overall mean age of patients was below 65 years of age to ensure the focus was on the elderly population, if patients had previous osteoporotic fractures, if studies focused on only the cost-reduction aspect of FLS, or if virtual FLS services were utilized. Two reviewers completed a blinded review process of the articles to decide on their inclusion or exclusion based on the determined criteria, and a third reviewer was used to break any ties. Quality assessment of the included studies was conducted using the Joanna Briggs Institute Critical Appraisal Tools, with studies categorized as low (>70%), moderate (50-70%), or high (<50%) risk of bias; discrepancies were resolved through discussion or consultation. All papers met the inclusion criteria, and the Preferred Reporting Items for Systematic Reviews and Meta-Analyses (PRISMA) guidelines were followed and used to develop a flow diagram of the selection criteria for reproducibility (Figure 1).

Results

Study Characteristics

Out of the 49 articles reviewed, most were categorized as retrospective cohort studies (n=20), followed by prospective cohort studies (n=13). There were also five prospective observational studies, three RCTs, two pre-post intervention studies, and two historical cohort studies. The remaining study designs included a population-based longitudinal study, a pilot single-center study, and one retrospective cohort study. The total number of patients included across all studies was 108,078, with female participants significantly outnumbering males.

Table 1 shows the studies reviewed, including the number of patients, secondary/recurrent fracture risk, anti-fracture medication initiation, and key findings. In 47 of 49 studies reporting sex, there were a total of 89,034 patients, including 77% females (68,588) and 23% males (20,446). The sample sizes varied widely across studies, from smaller cohorts (n=89) to large-scale registries (n=33,152). The mean for all studies was 2,205.67 participants. The breakdown by gender across studies was consistent. For example, one study included 16,086 females and 4,997 males [25]. The only exception was a large study with 30,433 males and 2,719 females [26]. The predominance of female participants reflects the higher prevalence of osteoporosis-related fractures among older women [27]. Mean ages consistently fell within 71-87 years of age, with the oldest cohort having an average age of 87 years.

**Table 1:** Impact of Fracture Liaison Services on Osteoporotic Fracture Outcomes in Older Adults

Author	Type of Study	Patients (M/F)	Mean age	Fracture location	# of FLS appointments	Follow Up Frequency/Duration	Secondary/Recurrent Fracture Risk	Anti-fracture medication initiation (% and med)	Conclusion
González-Quevedo et al. (2020) [1]	Prospective Cohort Study	724 (147 M, 577 F)	82.17	Not mentioned	3 FLS appointments in 1 year (including initial appointment)	12 months	No difference in risk between pre-FLS (3.6%) and FLS groups (4.6%) (p = 0.50)	Anti-fracture drug initiation: Pre-FLS: 12.3%, Post-FLS: 74.9% (p < 0.01)	FLS patients showed no significant difference in 1-year mortality or second fracture risk, but anti-osteoporotic drugs in FLS reduced mortality compared to pre-FLS.
González-Quevedo et al. (2022) [28]	Prospective Cohort Study	1101 (239 M, 862 F)	82.43	Femoral neck, trochanteric, subtrochanteric	4 FLS appointments in 1 year (including initial appointment)	1 month, 6 months, 12 months	Before FLS: 7.3%; After FLS: 6.6% (p = 0.65)	Total anti-osteoporotic drug prescription: 78.4% post-FLS vs 12.3% pre-FLS	FLS increased drug prescription and adherence, improved survival, but did not reduce second fracture risk.
Hawley et al. (2016) [26]	Population-Based Longitudinal Study	33152 (30433 M, 2719 F)	82.9	Hip	Not mentioned	Frequency is dependent on patient conditions	Not mentioned	Not mentioned	Orthogeriatric and FLS models improved post-hip fracture mortality.
Huntjens et al. (2014) [29]	Retrospective Cohort Study	1412 (378 M, 1034 F)	71.1	Nonvertebral	2 FLS appointments in 2 years (including initial appointment)	Not mentioned	Lower nonvertebral fracture risk in FLS group (Hazard Ratio 0.84 at 12 months, 0.44 at 24 months)	Not mentioned	FLS patients had 35% lower mortality and 56% lower nonvertebral fracture risk over 2 years.
Montoya-Garcia et al. (2022) [30]	Prospective Cohort Study	2965 (569 M, 2396 F)	82	Hip, spine, wrist, humerus	2 FLS appointments, with a median follow-up time being 12.3 months (including initial appointment)	Follow-up visits between 6-12 months after the first assessment, annually thereafter	Not mentioned	FLS-1 (inpatient): denosumab (49%), zoledronic acid (25.4%), alendronate (18.8%), teriparatide (6.2%), and risedronate (0.5%). FLS-2 (outpatient): alendronate (46.3%), denosumab (29.2%), teriparatide (8.9%), risedronate (7.6%), zoledronic acid (7.5%)	Spanish FLS units showed satisfactory quality indicators, but higher DXA use is needed.
Kikuchi et al. (2023) [31]	Pre-Post Intervention Study	251 (61 M, 190 F)	83.3	Proximal femoral fracture, vertebral	Not mentioned	Not mentioned	Not mentioned	53.3% initiated anti-fracture medications (bisphosphonates, denosumab, romosozumab, teriparatide, vitamin D, raloxifene)	FLS with screening and education effectively identified osteoporosis and initiated medication.
Lai et al. (2023) [32]	Pilot Single-Center Study	89 (32 M, 57 F)	79	Wrist, vertebral body, and hip	4 FLS appointments in 1 year (including initial appointment)	Telephone follow-ups at 1 month, 4 months, 12 months	6.25% of patients had a re-fracture in the non-FLS group vs. 2.44% of patients in the FLS group	Calcium and vitamin D daily, supplemented with zoledronic acid or alendronate	Combining FLS with online home nursing care improves monitoring, reduces falls, and enhances adherence.
Lawrence et al. (2017) [33]	Historical Cohort Study	7644 (953 M, 6691 F)	67.5	Not mentioned	Not mentioned	Not mentioned	Not mentioned	Bisphosphonates (oral/IV), denosumab, teriparatide	FLS increased intermediate osteoporosis outcomes in US veterans; more research on adherence and cost-effectiveness is needed.
Lüthje et al. (2021) [34]	Prospective Study	525 (119 M, 406 F)	74.85	Wrist, hip or proximal humerus	4 FLS appointments in 1 year (including initial appointment)	Not mentioned	Not mentioned	Denosumab (47.5%), bisphosphonates (oral) (38%), zoledronic acid (13.5%), strontium ranelate (1%), teriparatide (1%)	FLS reduced low-energy fractures in patients
Merle et al. (2017) [35]	Randomized controlled trial	436 F	67	Distal radius/ulna or proximal humerus	4 FLS appointments in 2 months (including initial appointment)	Not mentioned	Not mentioned	Not mentioned	Patient-centered care with a case manager improved post-fracture BMD investigation.
Muraoka et al. (2024) [36]	Retrospective Cohort Study	434 (98 M, 336 F)	86.8	Hip	6 FLS appointments in 2 years (including initial appointment)	24 months	Not mentioned	Not mentioned	FLS reduced hospital stay (p< 0.001), complications after admission (p< 0.001), and lowered secondary hip fractures, with lower mortality at 12 and 24 months.
Nakayama et al. (2016) [37]	Historical Cohort Study	931 (237 M, 694 F)	76.6	Hip	Not mentioned	Not mentioned	At 3-year follow-up, FLS hospital refracture rate was 16% compared to ~11% at non-FLS hospital (sub-distribution hazard ratio (SHR) = 0.67, confidence interval (CI) 0.47-0.95, p = 0.025	Not mentioned	FLS reduced re-fractures by 30% and major re-fractures by 40%; 20 patients needed to be treated to prevent one fracture over 3 years.
Naranjo et al. (2015) [38]	Observational Longitudinal Prospective Study	759 (162 M, 597 F)	72	Femoral head or neck, forearm, humerus	5 FLS appointments in 2 years (including initial appointment)	3 months, 6 months, 12 months, 24 months	Not mentioned	72% prescribed antiresorptive therapy. Medications: alendronate (49%), risedronate (27%), denosumab (17%), others (6%)	The Spanish program for secondary fracture prevention improved patient recruitment and treatment adherence.
Nassar et al. (2014) [39]	Prospective Cohort Study	362 (82 M, 280 F)	74.3	Vertebral	Not mentioned	Not mentioned	Not mentioned	Not mentioned	Detection of patients with vertebral fracture is similar for aBMD and TBS in patients with non-vertebral fractures. In patients with aBMD in the non-osteoporotic range, TBS adds information to lumbar spine aBMD alone and is related to an index of spine deterioration.
Olenginski et al. (2015) [40]	Retrospective Cohort Study	1088 (225 M, 863 F)	73.95	Hip, spine, wrist, vertebrae, pelvis, distal femur, periprosthetic, wrist, midshaft femur, subtrochanter femur	8 FLS appointments in 2 years (including initial appointment)	3 months, 6 months, 12 months, 18 months, 24 months	Not mentioned	Outpatient: zoledronic acid (44.2%), bisphosphonates (oral) (36.1%), teriparatide (4.1%), denosumab (2.9%), others (1.7%); Inpatient: bisphosphonates (oral) (52%), zoledronic acid (35.3%), teriparatide (6.6%), denosumab (4.4%), others (1.7%)	The outpatient and inpatient high-risk osteoporosis clinic (HiROC) model described here effectively and efficiently stratified and treated high-risk fracture patients.
Pennestrì et al. (2019) [41]	Observational Study	1278 - sex not specified	77.6	Proximal fracture of the femur and the humerus	Not mentioned	Not mentioned	0.24% of FLS patients had refractures, while 1.73% of patients who did not attend any FLS visits or exams had refractures	Not mentioned	Implementing an FLS in a high-volume orthopedic hospital effectively reduced secondary fractures, with only 0.24% of compliant patients experiencing another fracture compared to 1.73% among non-compliant patients.
Pflimlin et al. (2019) [42]	Retrospective Observational Study	256 (60 M, 196 F)	74.3	Hip, vertebral	Not mentioned	Not mentioned	Not mentioned	94.9% anti-fracture medication. Medications: zoledronic acid (60.2%), teriparatide (16%), denosumab (13.7%), bisphosphonates (5.3%)	FLS improved secondary fracture prevention, but patient identification and assessment timing need improvement.
Ruggiero et al. (2022) [43]	Prospective Observational Study	762 (187 M, 575 F)	83.5	Hip	4 FLS appointments in 1 year (including initial appointment)	3 months, 6 months, 12 months	The FLS group had a 19% refracture rate compared to 34.8% in usual care (p=0.0399)	75.1% of the FLS group vs 8.0% of the usual care group initiated anti-fracture medications (p < 0.0001). Medications: vitamin D/calcium (97.6%), complete therapy (63.7%)	FLS improved secondary prevention adherence, reduced hospital admissions, and improved survival.
Ruggiero et al. (2015) [44]	Prospective Observational Study	382 (82 M, 300 F)	83	Femoral neck and subtrochanteric	3 FLS appointments in 1 year (including initial appointment)	12 months	Not mentioned	Post-FLS: 48.51% received anti-fracture medications at discharge vs 17.1% pre-FLS (p<0.0001)	FPS improved the identification of high-risk patients, treatment adherence, and fall/fracture evaluations.
Sanli et al. (2019) [45]	Observational Cohort Study	282 (50 M, 232 F)	87	Hip, radius, ulna	Not mentioned	24 months	19% in both FLS attenders and non-attenders (p=1.0)	18% were started on anti-fracture treatment (74.5% with calcium, vitamin D, bisphosphonates; 25% with strontium)	FLS did not reduce fracture risk in patients >85 but lowered mortality and improved treatment adherence.
Scholten et al. (2020) [46]	Retrospective Chart Review	2097 (340 M, 1757 F)	73	Femur, hip, vertebral, humerus, radius, tibia, ankle, pelvis	Not mentioned	1 month, 12 months	1.05% on treatment had secondary fractures	76.96% initiated treatment. Medications: teriparatide (80.77%), denosumab (70.55%), alendronate (100%), zoledronic acid (56.25%), raloxifene (100%)	FLS improved adherence and reduced mortality in >85 of the cohort but did not reduce secondary fractures.
Silva et al. (2024) [47]	Retrospective Cohort Study	551 (91 M, 460 F)	80.83	Hip, vertebral, wrist, lower leg	Not mentioned	Over 36 months	Before FLS (13.3%) versus after FLS (5.9%) (Hazard Ratio 0.39; p = 0.006)	After FLS, 77.6% prescribed anti-fracture treatment versus before FLS, 8.1% (p = 0.001). Medications: alendronate, risedronate, zoledronic acid, denosumab	FLS increased osteoporosis treatment and reduced secondary fractures but had no significant effect on mortality.
Ahmed et al. (2025) [48]	Retrospective Observational Study	124 (24 M, 100 F)	68.55	Wrist, ankle, humerus, radius, clavicle, scaphoid, hand, foot, pelvis, and vertebral	Not mentioned	Not mentioned	Not mentioned	48.8% on anti-fracture medications. Medications: alendronic acid (43.3%), risedronate (30%), zoledronic acid (15%), teriparatide (6.67%), strontium (1.67%)	FLS identified high-risk patients and delivered cost-effective secondary prevention.
Amphansap et al. (2020) [49]	Prospective Cohort Study	353 (93 M, 260 F)	78.9	Femoral neck, intertrochanteric, subtrochanteric, isolated greater or lesser trochanter	5 FLS appointments in 1 year (including initial appointment)	1 month, 3 months, 6 months, 12 months	2.93% in the FLS group had secondary fragility fractures within 1 year of follow-up vs. 30% before FLS was implemented (p=<0.001)	89.38% received anti-fracture medications. Medications: calcium/vitamin D (82.42%), denosumab (28.57%), bisphosphonate (5.49%), teriparatide (10.63%), others (2.57%)	FLS reduced secondary fractures (30%→2.93%), increased BMD testing (28.33%→85.84%), and raised osteoporosis treatment rates (40.8%→89.38%). Surgery time and hospital stays decreased; mortality fell (9.2%→5.95%, not significant).
Arcidiacono et al. (2024) [50]	Prospective Observational Study	250 (74 M, 176 F)	85	Hip	3 FLS appointments in 1 year (including initial appointment)	6 months, 12 months	Not mentioned	79% received anti-fracture treatment. Medications: bisphosphonates (oral/IV) (87.2%), denosumab (7.7%), anabolic treatments (5.1%)	Anti-fracture therapy non-use fell (60%→21%) indicating greater patient adherence, while vitamin D/calcium prescriptions rose (29.6%→81%)
Axelsson et al. (2020) [25]	Retrospective Cohort Study	21,083 (4,997 M, 16,086 F)	73.9	Hip, clinical spine, humerus, radius, and pelvis	Not mentioned	Mean 20.4 months	18% lower risk in FLS period compared to pre-FLS period (HR = 0.82, 95% confidence interval [CI] 0.73-0.92, p=0.001)	FLS hospitals' medications increased from 14.7% to 28.0% (p	FLS reduced recurrent fractures by 18% through increased osteoporosis medication use.
Bachour et al. (2017) [51]	Retrospective Comparative Study	198 (51 M, 147 F)	75.5	Hip, spine, femur, pelvis, humerus and minor fractures (classified as all other bones)	Not mentioned	Over 24 months	18% of patients had a secondary fracture before FLS implementation vs. 8.2 % of patients in the FLS group (p=0.004)	Post-FLS: 54.1% maintained treatment vs 26% pre-FLS (p < 0.001)	FLS cut re-fracture risk by 54%, doubled BMD testing (28%→65%) and treatment adherence (26%→54%). NNT: 10.2 patients.
Beaupre et al. (2020) [52]	Retrospective Cohort Study	1427 (425 M, 1,002 F)	76.6	Hip	5 FLS appointments in 1 year (including initial appointment)	3 months, 6 months, 9 months, 12 months	Not mentioned	43.9% initiated medication: Pre-FLS: 24.7% (p < 0.001)	FLS nearly doubled osteoporosis treatment (24.7%→43.9%) across 1,427 patients. Sustained benefits were observed over seven quarters.
Aubry-Rozier et al. (2018) [53]	Retrospective Cohort Study	606 (123 M, 483 F)	78.5	Hip, humerus, spine, pelvis, radius	6 FLS appointments over 5 years (1 appointment at 3 months, 4 yearly clinical/biological evaluations (at years 1, 2, 3, and 4), and 2-3 DXA/VFA assessments (at years 2 and 4)	3 months with DXA+VFA every 2 years	FLS group (5.4%) versus the control group (5.1%) (p = 0.80)	79.0% received anti-fracture therapy: zoledronate (42.5%), alendronate (15.3%), ibandronate (IV) (10%), teriparatide (6%), denosumab (4%)	FLS patients had better outcomes than GP-managed ones: more BMD testing (72% versus 26.5%), more osteoporosis medication (79% versus 38.8%), and fewer fractures (3% versus 5.1%)
Billups et al. (2024) [54]	Retrospective Cohort Study	704 (176 M, 528 F)	77.1	Hip, vertebral, wrist	Not mentioned	6 months	Not mentioned	After FLS: Medication initiation increased from 25% to 47% (p < 0.001) (p < 0.01)	In a large healthcare system, FLS increased osteoporosis treatment (25%→47%, 57% with pharmacist), and DXA scans (63%→76%)
Bogoch et al. (2017) [55]	Prospective Observational Cohort Study	2,191 (607 M, 1,584 F)	71.65	Proximal femur, distal radius, proximal humerus, vertebra	Not mentioned	Not mentioned	Not mentioned	Medication initiation after FLS: Increased from 25% to 73% (inpatients) (p < 0.001) and 22% to 52% (outpatients) (p < 0.01)	BMD testing rose (84% inpatients, 85% outpatients) and osteoporosis treatment (73% inpatients, 52% outpatients)
Chandran et al. (2013) [56]	Retrospective Cohort Study	287 (29 M, 258 F)	72	Hip, vertebral, wrist	7 FLS appointments in 2 years (including initial appointment)	6 follow-ups over 24 months	Not mentioned	Following FLS treatment, 97.4% were started on medications. Medications: bisphosphonates (oral) (92.5%), strontium (18.7%), teriparatide (2.67%), raloxifene (2.67%), zoledronic acid (6.41%)	With FLS, there was a 97.5% DXA evaluation and 72.8% medication adherence over two years
Chang et al. (2021) [57]	Retrospective Cohort Study	1,099 (233 M, 866 F)	76.2	Hip, vertebral, wrist, upper arm	4 FLS appointments in 1 year (including initial appointment)	4 months, 8 months, 12 months	Not mentioned	Before enrollment, 24.4% were taking anti-fracture medication. After FLS initiation, 88.3% of the entire cohort was taking anti-fracture medications. At 12 months, 85.7% of the entire cohort was taking osteoporosis medications. Medications: denosumab (47-48%), alendronate (11-17%), zoledronic acid (9-19%), raloxifene (8-9%), ibandronic acid (4-7%), teriparatide (0.8-1.6%), strontium ranelate (<0.2%)	FLS improved osteoporosis care: 91.9% medication adherence, higher calcium/protein intake, and reduced falls (47.7%→24.3%).
Chen et al. (2022) [58]	Prospective Cohort Study	227 (63 M, 164 F)	Pre-FLS: 82.98 Post-FLS: 80.67	Femoral neck, peritrochanteric	5 FLS appointments in 1 year (including initial appointment)	3 months, 6 months, 9 months, 12 months	1-yr refracture rate was 11.8% in the pre-FLS group versus 4.9% in the post-FLS group (p=0.048)	Before FLS: 24.4% on medication; After FLS: 88.3%; 12 months: 85.7%; Medications: denosumab (47-48%), alendronate (11-17%), zoledronic acid (9-19%); Pre-FLS: 22.8% on medications; Post-FLS: 72.3% (p=0.000); Medications: denosumab (62.3%), bisphosphonates (25.7%)	FLS increased osteoporosis treatment (22.8%→72.3%), lowered 1-year refractures (11.8%→4.9%) and mortality (17.9%→11.8%), and improved functional recovery.
Chotiyarnwong et al. (2023) [59]	Retrospective Cohort Study	489 (137 M, 352 F)	78.4	Femoral neck, intertrochanteric	Not mentioned	Discharge, 12 months, 36 months	1-year refracture rate: 1.6% 3-year refracture rate: 5.7%	1-year post-FLS: 40.6% on medication vs 13.4% at discharge; Medications: bisphosphonates (oral) (27.1%), denosumab (10%), bisphosphonates (IV) (2.4%), teriparatide (1.0%) and strontium ranelate (0.2%)	FLS improved BMD assessment (81.2%), calcium/vitamin D use (>90%), and maintained osteoporosis treatment (40%). Three-year mortality was 20.4%; refracture rate, 5.7%.
Cosman et al. (2017) [60]	Pre-Post Intervention Study	135 (35 M, 100 F)	Pre-FLS: 77.4 Post-FLS: 79.7	Hip	Not mentioned	4-6 months after discharge	Not mentioned	Post-FLS for hip fractures: 19% on medication vs 33% pre-FLS (p = NS); No hip fracture: 21% post-FLS vs 38% pre-FLS (p < 0.05)	FLS boosted vitamin D intake (48%→68%) and BMD testing (35%→65%), but osteoporosis medication use fell (33%→19%).
Delbar et al. (2021) [27]	Observational Retrospective Cohort Study	380 (79 M, 301 F)	76	Vertebral, hip, proximal humerus, pelvis, wrist/forearm	4 FLS appointments in 2 years for patients on teriparatide. 2 FLS appointments in 2 years for patients on zoledronic acid, denosumab, and oral bisphosphonates.	6 or 12 months, depending on treatment type	Not mentioned	After FLS: 84.1% on medication at 12 months vs 70.3% at 24 months (p < 0.05); Medications: zoledronic acid (54.5%), teriparatide (22.9%)	FLS improved osteoporosis treatment persistence (12 months: 84.1%, 24 months: 70.3%). Denosumab had better adherence than oral bisphosphonates.
Eekman et al. (2014) [61]	Prospective Observational Study	2,207 (536 M, 1,671 F)	70	Wrist, hip, humerus, ankle, vertebral	5 FLS appointments in 1 year (including initial appointment)	3 months, 6 months, 9 months, 12 months	2.1% had subsequent fractures within 1 year	After FLS: 50.6% responded to screening vs 5% pre-FLS; Medications: bisphosphonates (oral) (90%), strontium (4%), calcium/vitamin D (3%)	FLS increased osteoporosis screening (51%), treatment retention (88% at 1 year), and reduced new fractures (2%).
Galasso et al. (2023) [62]	Retrospective Cohort Study	320 - sex not specified	74.83	Femoral neck, peritrochanteric, proximal/distal femur, periprosthetic, acetabulum/pelvis, ankle	Not mentioned	Not mentioned	Before FLS (8.0%), after FLS treatment (2.4%) (p < 0.026)	56.7% initiated treatment: Pre-FLS: 16.1% (p < 0.001)	FLS boosted DXA scans (25.9%→58.2%), treatment initiation (16.1%→56.7%), and cut 2-year re-fractures (8.0%→2.4%).
Gonzalez-Quevedo et al. (2024) [63]	Prospective Cohort Study	1,366 (432 M, 934 F)	82.34	Femoral neck, trochanteric, subtrochanteric	3 FLS appointments in 1 year	1 month, 6 months, 12 months	Pre-FLS (10.5%) versus Post-FLS (10.0%) (p = 0.78)	79.3% initiated treatment: Pre-FLS: 12.5% (p < 0.01); Medications: bisphosphonates (oral) (83.8%), denosumab (7.6%), teriparatide (8.6%)	FLS increased osteoporosis prescriptions (12.5%→79.3%), treatment adherence (30.2%→51.7%), and lowered adjusted 1-year mortality.
Singh et al. (2019) [64]	Prospective Controlled Study	195 (31 M, 164 F)	70.5	Wrist, humerus, hip, vertebral, rib, pelvis, foot, ankle	3 FLS appointments in 1 year (including initial appointment)	6 months	FLS: 3.0%, Control: 1.8% (p=0.664)	Post-FLS: 77.8% high-risk patients on treatment vs 22.9% pre-FLS (p < 0.001)	In the FLS group, 77.8% of high-risk patients received appropriate treatment, compared to only 22.9% in the usual care group. The FLS program demonstrated increased rates of bone density testing, specialist referrals, and blood testing, as well as improved health-related quality of life.
Sorensen et al. (2021) [65]	Retrospective Cohort Study	137 (54 M, 83 F)	72.4	Lumbar, thoracic, lumbar and thoracic, sacral	Not mentioned	12 months	Preventable fractures: 8.8% in the treatment group versus 23.3% in the untreated group	After FLS (vertebroplasty): 73.0% on treatment vs 15% pre-FLS	Only 15% of patients were initially treated, but the FLS increased bone density testing from 17.5% to 68.4% and vitamin D screenings from 16.4% to 73.7%, p<0.001 for both.
Sujic et al. (2020) [66]	Retrospective Cohort Study	6543 (1168 M, 5375 F)	68.3	Distal radius, ankle, proximal humerus, hip, multiple fractures, elbow, spine, pelvis, tibia/fibula, clavicle	Not mentioned	60 months	Not mentioned	Not mentioned	FLS helps reduce mortality by identifying high-risk patients, particularly those with multiple fractures, hip or proximal humerus fractures, and older age. By targeting these groups, FLS improves survival outcomes through focused interventions and better risk stratification, with lower mortality rates over time.
Valladales-Restrepo et al. (2023) [67]	Retrospective Cohort Study	438 (94 M, 344 F)	77.5	Hip, vertebral, radius/ulna, humerus, tibia/fibula	The FLS followed up with 438 patients initially at an average of 83 days after discharge, with 251 patients (57.3%) receiving a second visit at an average of 228 days after discharge, 170 patients (38.8%) receiving a third visit at an average of 407 days after discharge, and 109 patients (24.9%) receiving a fourth visit at an average of 578 days after discharge, over a total follow-up period of 682.9 days.	22.4 months	3.7% experienced new fractures	After FLS: 50.7% initiated therapy versus 16.7% pre-FLS (p = 0.396); Medications: teriparatide (21.2%), denosumab (16.4%), zoledronic acid (18%), bisphosphonates (oral) (0.9%)	FLS had an effectiveness rate of 73.6%. The program successfully prevented new fractures, with only 3.7% of patients experiencing new fractures during follow-up.
Vranken et al. (2022) [68]	Retrospective Cohort Study	8,682 (2,606 M, 6,076 F)	68.2	Hip	Not mentioned	36 months	Major/hip fractures: 6.0% pre-FLS versus 5.6% post-FLS (p=0.616). Non-major/non-hip fractures: 3.3% pre-FLS versus3.2% post-FLS (p=0.852)	40% initiated medication: Pre-FLS: 12.6% (p < 0.001); Medications: Aaendronate (25%), zoledronic acid (5%), denosumab (7%), teriparatide (2%), calcium supplements (3%), vitamin D supplements (4%), and hormonal treatments (1%)	FLS has two main benefits for patients with major/hip fractures: a 16% lower mortality risk over 3 years and a 33% lower risk of subsequent major/hip fractures in the first year after the initial fracture. However, for patients with less severe (non-major/non-hip) fractures, the FLS program showed no significant benefits in either mortality or subsequent fracture rates. These results suggest that FLS programs are most effective for patients with serious fractures.
Vrignaud et al. (2018) [69]	Retrospective Observational Cohort Study	247 (19 M, 228 F)	68.7	Wrist, hip, vertebral, ankle, upper humerus	Not mentioned	Not mentioned	Not mentioned	Post-FLS: 57.5% on medications vs 48.1% pre-FLS (p = 0.24); Medications: bisphosphonates (oral) (14.2%), bisphosphonates (IV) (36.8%), denosumab, teriparatide, raloxifene (6.5%), vitamin D (52%)	FLS demonstrated improvements in osteoporosis prevention, with decreases in family history of hip fractures (22.2% to 10.9%), vertebral fractures (37% to 20.2%), and vitamin D deficiency (83.3% to 71.8%). The program primarily served women (92.3%) with an average age of 68.7 years.
Wang et al. (2023) [70]	Prospective Cohort Study	556 (179 M, 377 F)	79.8	Femoral neck, trochanteric, subtrochanteric	Not mentioned	12 months	1-year subsequent fracture rate: control (4.9%) versus FLS group (4.4%) (p=0.752). Secondary hip fracture: control (3.9% ) versus FLS group (2.4%) (p=0.299).	After FLS implementation (73.4%) versus before FLS (35.5%) received anti-osteoporotic treatment (p <0.001)	FLS implementation improved time to surgery (98% versus 91.7%) and reduced hospital stays (8.1 versus 9.8 days). Patients receiving anti-osteoporotic treatment through FLS had significantly lower mortality (9.7% versus 20.9%) compared to untreated patients.
Wong et al. (2021) [71]	Retrospective Observational Study	226 (38 M, 188 F)	77.3	Vertebral compression fractures (T1-L5)	5 FLS appointments in 2 years (including initial appointment)	6 months, 12 months, 18 months, 24 months	0.4% had an imminent fracture within 1 year	100% prescribed denosumab injections	With FLS, only 0.4% of 226 patients had a subsequent fracture within 2 years, with 89.8% adhering to treatment. While falls occurred in 11.1% of patients and mortality was 7.1%, the program demonstrated effectiveness in fracture prevention, though muscle strength assessments are recommended for future improvement.
Zinger et al. (2021) [72]	Randomized Controlled Study	127 (29 M, 98 F)	79.2	Femoral neck, interchanteric, subcapital, subtrochanteric	4 FLS appointments in 19 months (1.58 years)	Every 4 months	Not mentioned	After FLS, 100% initiated anti-osteoporotic medication (vs 0% before, p < 0.001). Medications: Vitamin D (100%), calcium (100%), thyroid (9.4%), hyperparathyroidism (3.9%), modified Vitamin D for renal failure (8.3%)	The FLS program proved highly effective in identifying and addressing underlying health issues in patients with hip fragility fractures. By combining FRAX and DEXA screening tools, researchers could predict fracture risks with 93% accuracy and discovered clinically significant medical conditions in 27% of patients. The study underscores the importance of comprehensive fracture prevention programs in improving patient care and reducing future fracture risks.

Fracture locations were primarily hip-related (i.e., femoral neck and trochanteric), though vertebral, wrist, and humerus fractures were also common. Some studies, including Wong et al. [71], exclusively examined vertebral fractures, while others, such as Bachour et al. [51], included a broader range of fracture sites and categorized fractures as "major" or "minor". Notably, hip fractures were the most frequently studied, likely due to their high morbidity and mortality [1,30].

Structure and Intensity of FLS Programs

FLS programs demonstrated variability in structure, particularly in appointment frequency and follow-up duration. Most studies reported two to five appointments within the first year, though some extended follow-up to three years. The most intensive program, described by Olenginski et al. [40], included eight appointments over two years [31], while others, such as Hawley et al. [26], did not specify appointment numbers, but emphasized condition-dependent follow-up [26].

Follow-up frequency ranged from monthly check-ins [32] to annual visits [1]. Hybrid models, such as a combination of in-person appointments and telephone check-ins, were also noted [33]. Duration of follow-up correlated with study aims: shorter-term studies (i.e., six months in Billups et al. [54] assessed immediate treatment initiation, while longer-term studies (i.e., three-year analysis in Vranken et al. [68]) evaluated mortality and refracture risk.

Across the literature, the implementation of FLS demonstrated variable, but consistently positive impacts on secondary fracture rates.

Impact on Refracture Risk

Of the 20 of the 49 studies that compared refracture rates between FLS and non-FLS groups, most reported improvements, though only 11 of the 20 manuscripts that report p-values found statistically significant differences. Reported reductions in refracture risk ranged widely, from 4.9% post-FLS versus 12.5% with usual care, to 2.9% versus 30% at one-year follow-up without FLS. The most pronounced effect was seen in a prospective study, where refracture rates dropped from 34.8% among controls to 19% in the FLS cohort (p = 0.0399) [36], suggesting that program effectiveness may depend on baseline risk and the intensity of the intervention. The benefits of FLS were further supported by reductions in refracture rates. On average, absolute risk reductions ranged from 4.9% to 11.8%, while relative risk reductions ranged from 18% to 40%. Reported hazard ratios were as low as 0.82. This variability reflects differences in program design (ranging from two to eight FLS appointments over one to five years), follow-up durations (from monthly check-ins to annual visits for 60 months), and baseline patient risk.

Meanwhile, eight of the 20 studies found no fracture reduction benefits with FLS. In those studies, fracture rates in control versus FLS groups showed either marginal differences (i.e., 7.3% versus 6.6%; p = 0.65) [1] or identical outcomes (19% in both groups; p = 1.0) [37].

Impact on Medication Initiation

The implementation of FLS was consistently associated with increased initiation of anti-fracture medications, though the degree of improvement varied by study. Reported increases ranged from a 9.4% increase post-FLS to a 100% increase post-FLS. The studies show an average increase of 46.4% for anti-fracture medication usage post-FLS implementation. The most substantial impact was seen within the randomized study by Zinger et al. [72], in which 100% of patients received treatment after FLS involvement, highlighting the potential of structured programs to close treatment gaps. These findings suggest that, while FLS models vary in scope and intensity, they consistently improve medication initiation across settings.

Types of Medication Prescribed

The medications and/or supplements that were prescribed to patients included alendronate, risedronate, ibandronate, zolendronic acid, denosumab, teriparatide, romosozumab, raloxifene, strontium ranelate, vitamin D, and calcium. The most commonly prescribed medications were zoledronic acid, denosumab, and alendronate. Montoya-Garcia et al. [30] detailed differences between inpatient and outpatient prescriptions, with denosumab and alendronate being frequent choices [39]. Other studies, such as Kikuchi et al. [31], reported the use of a variety of pharmaceutical therapies, including additional agents such as romosozumab and raloxifene [40].

These studies showed that more patients started treatment after joining an FLS, highlighting how these programs can help close gaps in care and promote compliance with medications they need to prevent future fractures.

Discussion

Burden of Osteoporotic Fractures

Osteoporotic fractures represent a growing burden, particularly among aging postmenopausal women, where secondary fractures occur in up to 54% of patients within five years [73]. FLS programs have emerged across the country as a targeted strategy to help close care gaps. Our analysis showed that effectiveness varied across studies, depending on program structure and baseline patient characteristics.

Patient Demographics and Program Design

The effectiveness of FLS in reducing fracture risk was influenced by patient demographics and program design. Programs demonstrating the most significant reductions (i.e., 30%-56% lower refracture rates) typically targeted younger, higher-functioning cohorts with intensive follow-up (i.e., ≥4 annual visits) and multidisciplinary care components [1,33,74]. For instance, Lai et al. [32] reported a 60% reduction in refractures by combining FLS with nursing homes and telemedicine follow-ups, achieving 89% treatment adherence through personalized and frequent touchpoints [1,33,74]. Meanwhile, studies of older, frailer populations (mean age >85) often showed marginal benefits. Sanli et al. [45] found no refracture reduction (19% in both FLS and control groups; p = 1.0) among patients aged ≥87, likely due to comorbidities, polypharmacy, and lower treatment tolerance [1,35,37].

These differences were likely multifactorial. From a biological standpoint, aging is associated with reduced bone remodeling activity and a diminished response to common osteoporosis treatments such as bisphosphonates [75]. Behaviorally, older adults face additional challenges, including cognitive impairment, medication overload, and mobility limitations, all of which have been shown to lower adherence and reduce the effectiveness of secondary prevention programs [76]. Together, these factors suggest that FLS programs may require more tailored strategies to achieve similar benefits in very elderly or medically complex populations.

The differences in outcomes are likely due to a combination of biological and practical challenges faced by older adults. As people age, their bones tend to respond less effectively to treatment, and conditions such as cognitive decline, multiple medications, and limited mobility can make it harder to adhere to recommended care. One approach to addressing these challenges is the use of risk assessment tools such as FRAX scores and DXA scans. These tools help identify patients at the highest risk, including those with very low bone density or a high predicted risk of future fractures. By recognizing these individuals early, FLS programs can provide more focused and intensive interventions to improve outcomes in this vulnerable population [25].

Determinants of FLS Effectiveness

The effectiveness of FLS interventions has been shown to be influenced by three key factors: biological considerations, program intensity, and risk stratification. Accelerated bone loss among elderly patients with osteoporosis often requires alternative therapies such as teriparatide rather than standard bisphosphonates, as frailer patients show less favorable response to conventional treatments due to complex and frequent administration [77]. In patients with osteoporosis, treatment with teriparatide showed a greater increase in BMD at the lumbar spine and femoral neck compared to treatment with bisphosphonates [78]. Current osteoporosis medications have shown differences in re-fracture rates and side effects [79]. As such, variation in the types of medications used across FLS programs may contribute to differences in outcomes, potentially influencing adherence rates, fracture risk reduction, and overall program effectiveness. Program intensity, measured by the frequency of follow-ups, also impacts outcomes. Studies with monthly follow-ups [61] enhanced adherence to medications through early side-effect management, while less frequent, yearly follow-ups correlate with 16.4% higher medication discontinuation rates [27]. In addition, risk stratification using tools such as FRAX scores or DXA-driven protocols allowed clinicians to better identify patients who were at high risk for future fractures and prioritize them for more aggressive treatment strategies [25]. Effective risk stratification ensures that limited resources are directed toward those most likely to benefit, which may contribute to improved outcomes across FLS programs.

Clinical evidence reveals three critical, interconnected requirements for successful FLS implementation: Sustained follow-up duration, innovative care delivery models, and treatment adherence strategies.

Follow-Up Duration and Long-Term Outcomes

The duration of follow-up was demonstrated to be a key determinant in assessing mortality benefits. Short-term studies (≤1 year), including González-Quevedo et al. [63], found no difference in mortality or refracture rates. Meanwhile, when the follow-up duration was extended to 2+ years, the benefits became clear [1]. For instance, programs with longer follow-ups showed up to 35% improvement in survival rates and a 56% reduction in nonvertebral fractures [35,42]. This suggests that short-term studies may miss the long-term benefits, especially for patients with hip fractures, where the greatest reduction in mortality happens at approximately three years.

Telemedicine has been shown to revolutionize care delivery, especially for patients in rural areas. For instance, Lai et al. [32] found that a hybrid FLS program involving telephone follow-ups resulted in 90% adherence, as compared with 64% for the control group, and significantly reduced refracture rates (4.9% in the FLS group versus 12.5% in the control group) [33]. Similarly, Wang et al. [70] reported improvements in treatment uptake (73.4% in the FLS group versus 35.5% in the control group) and lower rates of mortality (9.7% in the FLS group versus 20.9% in the control group) in remote populations in Asia, where virtual visits eliminated the need for travel [72]. These examples suggest that flexible care models can address geographic barriers while improving patient outcomes.

Medication Initiation and Adherence

FLS programs have consistently demonstrated a positive impact on the initiation of anti-fracture medications following osteoporotic fractures. Implementation of FLS was associated with a substantial increase in treatment rates, though the degree of improvement varied. González-Quevedo et al. [63] reported an increase from 12.3% pre-FLS to 74.9% post-FLS [1]. In addition, Ruggiero et al. [43] found that 8% of patients in usual care initiated treatment compared to 75.1% in the FLS group (p < 0.0001) [36]. In other settings, initiation rates rose from 40.8% to 89.38% [32] and from 22.8% to 72.3% [3] following FLS implementation. The most striking improvement was observed in an RCT by Zinger et al. [72], where treatment initiation increased from 0% to 100% [38]. Even in studies with more modest gains, such as Vrignaud et al. [69] (48.1%-57.5%), FLS still contributed to closing the care gap. These findings suggest that FLS models can be effective for improving the timely initiation of osteoporosis medications across a wide range of healthcare settings and patient populations, which warrants further investigation in additional studies.

On average, 70%-90% of patients initiated anti-fracture treatment following FLS enrollment, with most studies reporting initiation within the first month after fracture, often at discharge or during the first follow-up visit. This trend aligns with previous research that suggests that the pre-discharge period following a fracture is the most effective time to begin a new intervention [80,81].

Adherence at one year was generally high, with some programs reporting rates above 85% [61], though longer-term adherence declined by up to 16% in certain programs [27]. Programs with more frequent follow-ups (i.e., ≥4 appointments/year) often reported higher medication adherence (up to 88.3%) and lower secondary fracture rates (4.9% versus 11.8% pre-FLS) [61]. Meanwhile, the variability in refracture risk reduction, ranging from 30%-56% reductions [42] to nonsignificant differences [37], suggests moderating factors such as follow-up rigor, patient risk stratification, and medication adherence. Notably, the most robust outcomes were tied to multidisciplinary FLS models incorporating nursing, pharmacy, or coordinated care teams, which correlated with higher DXA screening rates, sustained adherence, and reduced hospitalizations.

Value of Multidisciplinary and Structured Approaches

These findings advocate for structured, team-based approaches to optimize FLS efficacy. For example, Ruggiero et al. [43] showed that a coordinated approach between nursing and pharmacy reduced refractures from 34.8% to 19% and increased anti-osteoporosis drugs initiation to 75% from 8% (p < 0.0001) [36]. Similarly, a high-risk clinic model with a multidisciplinary approach achieved 97.5% DXA screening and 72.8% adherence over two years [59]. These results highlight the importance of structured, multidisciplinary teams and protocols such as (a) universal FLS with 4+ annual visits for high-risk patients (i.e., those with hip or vertebral fractures); (b) first-line therapies such as zoledronic acid or denosumab for elderly patients; and (c) hybrid models that combine telemedicine with in-person DXA screenings.

Collectively, these insights advocate for standardized, long-term FLS protocols with adaptable delivery methods to maximize fracture prevention and survival benefits across diverse settings, which warrant further investigation.

Early enrollment into an FLS program, ideally before hospital discharge, emerged as a critical factor for successful secondary fracture prevention. Several studies in this review reported initiating treatment at or immediately following discharge, which was associated with significantly higher medication initiation rates and improved outcomes [1,32,51]. Delays in treatment initiation were linked to lower adherence to anti-fracture medication and worse patient outcomes. Incorporating FLS enrollment into standard discharge planning ensured timely risk assessment, facilitated early pharmacologic treatment, and promoted continuity of care. Given the consistent benefits observed across multiple settings, further investigation is warranted, and the integration of FLS into inpatient workflows should be considered with further supportive evidence for patients presenting with osteoporotic fractures.

Study Limitations

This study has a few important limitations to consider. The findings were based on a review of several different published studies, each with its own methods, patient populations, and definitions of outcomes. Based on these differences, it is difficult to directly compare the effectiveness of one FLS program to another. Some studies did not provide enough detail about when medications were started, how long patients stayed on treatment, or how often they were followed up, which limits the ability to conclude what aspects of FLS design mattered most. In addition, most of the studies included were observational, meaning patients were not randomly assigned to FLS or non-FLS groups. This makes it possible that other factors, such as a patient’s overall health, motivation, or access to care, could have influenced the results. Furthermore, this review relied on published data. As such, there was no access to individual patient information, which means personal factors such as other medical conditions or socioeconomic status, which may affect treatment outcomes, were not accounted for. These limitations highlight the need for additional, larger-scale RCTs, as well as more standardized reporting to gain insight into FLS components that are most effective.

## Conclusions

Adoption of an FLS into patient care offers a structured approach to improve outcomes following osteoporotic fractures and reduce the risk of future injury. Across the 49 studies reviewed, FLS implementation was consistently associated with higher rates of starting anti-fracture medication and often led to significant reductions in secondary fracture rates. These findings reflect the importance of structured, multidisciplinary programs, which include timely follow-up, coordinated care, and patient education.

While outcomes varied depending on program design and patient population, early enrollment, especially before discharge, stood out as a key factor for improving long-term outcomes. Importantly, not all studies demonstrated uniformly positive outcomes, suggesting that program effectiveness may depend on patient characteristics, such as age or baseline fracture risk, as well as system-level factors such as follow-up duration and resource intensity. This variability underscores the need for careful consideration of which patients and settings are most likely to benefit, rather than a one-size-fits-all approach. Osteoporotic fractures and undertreatment increase the risk of secondary fractures and hospitalizations. Further studies are needed to evaluate how FLS can be integrated effectively into clinical care. Future research should also explore which program components, including follow-up intensity, the use of dedicated coordinators, and primary care integration, drive the greatest improvements in patient outcomes. Clinically, these findings highlight both the promise of FLS models and the importance of tailoring their implementation to maximize benefit while acknowledging areas of uncertainty.
